# Supplementary material for: The Associations between 24-Hour Movement Behaviours and Quality of Life in Preschoolers: A Compositional Analysis of Cross-Sectional Data from 2018–2021
Source: Int J Environ Res Public Health. 2022 Nov 14;19(22):14969. doi: 10.3390/ijerph192214969 (PMC9690870; doi:10.3390/ijerph192214969)
Supplement: Supplementary file 1 [file ijerph-19-14969-s001.zip › Supplementary Material S1.pdf]

Supplementary Material S1

1. Classification of Activity Codes into PA, SB and SL

Table S1. Classification of Activity Codes into PA, SB and SL

Activity code	Classification
Doing craftworks<br>（Drawing/ painting/ sketching on paper/other forms of craftwork）	SB
Reading printed book	SB
Electronic device use<br>(for Learning, for entertainment, for media creation for communication and other screen-based activities）	SB
Simple household chore	PA
Indoor play<br>(dancing, crawling, playing board games, playing with a manipulative toy）	PA
Outdoor play<br>(playing ‘hide-and-seek’ / ‘catching’ in a playground/other play)	PA
Afternoon naps	SL
Sleep in night	SL

PA = Physical Acivity; SB = Sedentary Behaviour; SL = Sleep.

2. Data filtering principles

The sleep duration category for different age groups was defined according to sleep duration recommendations from WHO Guidelines on physical activity, sedentary behavior and sleep for children under 5 years of age[1] and the American Academy of Sleep Medicine[2]. For children aged 1-2 years and children aged 3-4 years, the WHO sleep（SL）duration standards are the same as those of the AASM. Hence, the SL duration range for children aged 2 years determined in this study was 11-14 hours and for children aged 3-4 years was 10-13 hours. The WHO guidelines do not have recommendations for children of age 5 and above while the AASM recommends 10-13 hours for children aged 5 years and 9-12 hours for children of ages 6 and above. As such, the SL duration range determined in this study for children aged 5-6 years is 9-12 hours. Taking the variability and fluctuation of time into consideration, sleep duration± 2 hours of the age-specific sleep range were included in the data analysis.

Because children sleep for longer periods of time during the day, parents tend to report SL time more accurately than physical activity (PA) and sedentary behavior(SB) time, so the filtering principles for PA and SB time were further determined on the basis of SL time. As mentioned in the main text, the sum time of PA, SB and SL is 24 hours. The time outside of SL may be both PA and SB, so we subtract the baseline time of SL for each age group from 24 hours, which is the PA or SB time. For example, if a 2-year-old child sleeps 9-16h, the upper limit of PA or SB time is 24h-9h=15h.

It is important to note that the children could not have no PA or SB during the day, so the total time of PA and total time of SB were both set to greater than 0. For the content of the subordinate dimensions of SB and total time of PA, the corresponding time of specific content such as studying, doing handicraft, and taking nap was null indicated because some children did not participate in this activity and so was defaulted to 0 value. For night SL, as the null value did not exceed 5%, it was replaced by the mean value of the corresponding year and age group.

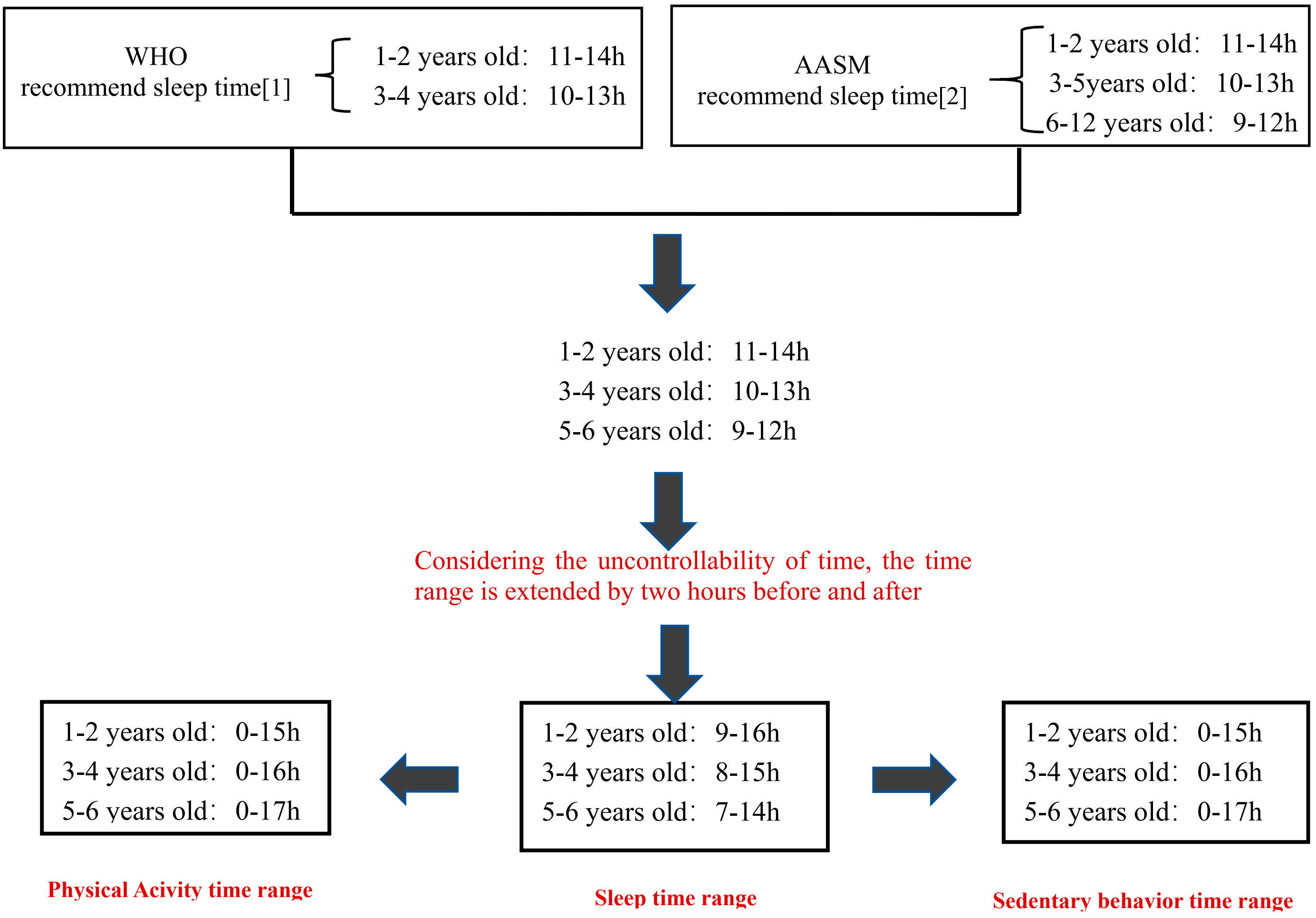

3. Study participants and data cleaning process

Based on the data filtering criteria described above, all data from 2018-2021 were sorted, and the data of 8045 participants were finally retained. Please refer to Figure S1 for more details.

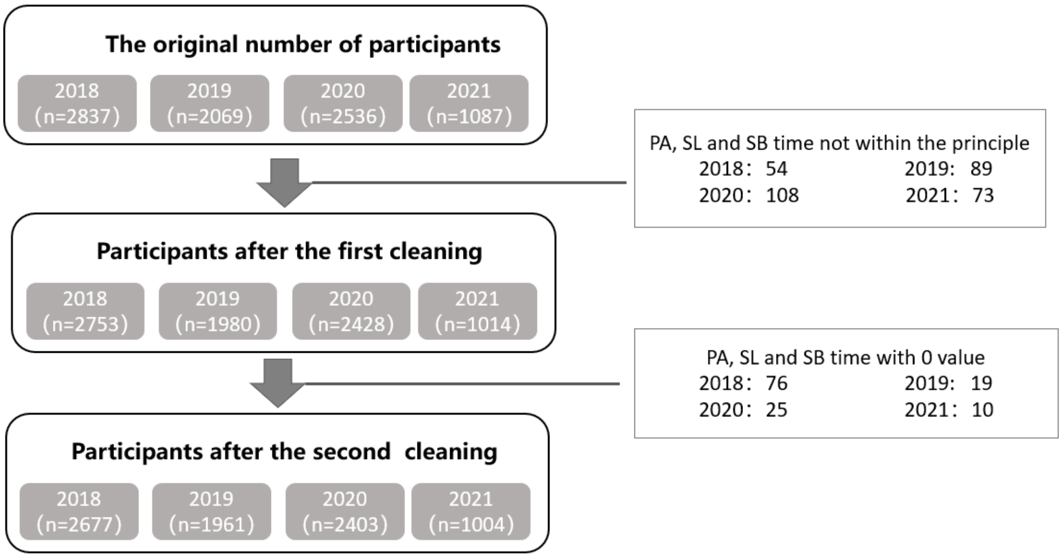

Figure S1 Flow chart and break down of number of survey respondents after data cleaning

Reference:

1. World Health Organization *Guidelines on physical activity, sedentary behaviour and sleep for children under 5 years of age*; World Health Organization: Geneva, 2019; ISBN 978-92-4-155053-6.
2. Paruthi, S.; Brooks, L.J.; D'Ambrosio, C.; Hall, W.A.; Kotagal, S.; Lloyd, R.M.; Malow, B.A.; Maski, K.; Nichols, C.; Quan, S.F.; et al. Recommended Amount of Sleep for Pediatric Populations: A Consensus Statement of the American Academy of Sleep Medicine. *Journal of Clinical Sleep Medicine* **2016**, *12*, 785–786, doi:10.5664/jcsm.5866.
