# Supplementary material for: The Associations between 24-Hour Movement Behaviours and Quality of Life in Preschoolers: A Compositional Analysis of Cross-Sectional Data from 2018–2021
Source: Int J Environ Res Public Health. 2022 Nov 14;19(22):14969. doi: 10.3390/ijerph192214969 (PMC9690870; doi:10.3390/ijerph192214969)
Supplement: Supplementary file 1 [file ijerph-19-14969-s001.zip › Supplementary Material S2.pdf]

Supplementary Material S2

1 Linear regression model

We express the three-parts daily activity which consists: sleep(SL), sedentary behavior(SB), physical activity(PA) as a set of isometric log-ratio(ilr) coordinates:[z_{i1}, z_{i2}], and build our multiple linear regression model with the outcomes of quality of life（QoL）scores and the ilr coordinates as follows.

$$zQoL_i = \beta_0 + \beta_1 z_{i1} + \beta_2 z_{i2} + covariates_i ,$$

$$z_{i1} = \sqrt{\frac{2}{3}} ln(\frac{SL_i}{\sqrt{SB_i \times PA_i}}), \quad \text{(S1)}$$

$$z_{i2} = \sqrt{\frac{1}{2}} ln(\frac{SB_i}{PA_i}),$$

The coefficient β_1 corresponds to z_{i1}, which is the log-ratio of SL_i, to the compositional mean of the other behaviors (SB_i, PA_i). Then with premuting the composition iteratively, we place each behavior to the first place to get another 2 compositions and apply the new composition to the above ilr coordinates to get the different linear models.

$$composition1 = [SL_i, SB_i, PA_i]$$
$$composition2 = [SB_i, PA_i, SL_i] \quad \text{(S2)}$$
$$composition3 = [PA_i, SL_i, SB_i]$$

2 Isotemporal substitution model

With composition1 we have our predictive model:

$$zQoL_i = \beta_0 + \beta_1 z_{i1} + \beta_2 z_{i2} + covariates_i \quad \text{(S3)}$$

the ilr coordinates are defined above.

Next we need to estimate the baseline of predictive quality of life from the model S3 we have above. Then using the geometric mean of composition1($[\overline{SL_i}, \overline{SB_i}, \overline{PA_i}]$, here the label $\overline{X}$ is geometric mean of X) to calculate the ilr coordinate and get the estimate of baseline: $\widehat{\overline{zQoL}}$

After that, we estimate $\widehat{zQ}$ for new composition where a fixed duration of time has been reallocated from one behaviour to another, keeping the remaining behaviours constant. For example, when 30 minutes are reallocated from SL to SB, the new composition is:[$\overline{SL} - 30, \overline{SB} + 30, PA$], then we ilr transform this new composition to get the new [z'_{i1}, z'_{i2}], and estimate Q as follows:

$$\widehat{zQoL}_{(-30,+30,0)} = \beta_0 + \beta_1 z'_{i1} + \beta_2 z'_{i2} + covirates_i \quad \text{(S4)}$$

Finally, the expected difference in total-life-quality between a 30 minutes reallocated composition and the mean composition is:

$$\widehat{zQoL}_{(-30,+30,0)} - \widehat{\overline{zQoL}}$$

we can use this approach to estimate any reallocation between compositional parts.
