# Supplementary material for: The Associations between 24-Hour Movement Behaviours and Quality of Life in Preschoolers: A Compositional Analysis of Cross-Sectional Data from 2018–2021
Source: Int J Environ Res Public Health. 2022 Nov 14;19(22):14969. doi: 10.3390/ijerph192214969 (PMC9690870; doi:10.3390/ijerph192214969)
Supplement: Supplementary file 1 [file ijerph-19-14969-s001.zip › Supplementary Material S3_Sample SMALLQ & PedsQL.pdf]

www.nie.edu.sg
1 Nanyang Walk Singapore 637616 Tel: (65) 6790 3888
NTU Reg No. 200604393R

SMALLQ®

SURVEILLANCE OF DIGITAL MEDIA HABITS IN EARLY CHILDHOOD QUESTIONNAIRE

(Outside of the preschool)

Survey Instructions

1. This survey is to be completed by **parents of preschool children aged 2 to 6 years**.
2. It will take about 20 minutes to complete.
3. Your answers are very important to us. There are no right or wrong answers.
4. If you have **more than a child in the centre**, please answer in relation to only **one child (i.e. Select only 1 child).**
5. This survey will be repeated 12 and 24 months from now.

How much time does your child spend in preschool or kindergarten each weekday on an average?

☐ Less than 3 hours
☐ 3 to 5 hours
☐ More than 5 hours but less than 8 hours
☐ More than 8 hours
☐ Not applicable

This survey classifies digital media into six categories.

1) Television
2) Computer (e.g. desktops, laptops)
3) Mobile devices (e.g. smartphones, tablets)
4) Video game devices (e.g. console and handheld gaming players, virtual reality headset)
5) Blue-ray/DVD/CD/Videotape players
6) Intelligent/ technology toys (e.g. programmable toys)

SMALLQ® is trademarked in 2018 under Class 41 TM120494
c/o Professor Michael Chia, National Institute of Education, Nanyang Technological University, Singapore.

www.nie.edu.sg
1 Nanyang Walk Singapore 637616 Tel: (65) 6790 3888
NTU Reg No. 200604393R

Segment I: Digital media use

Digital environment at HOME

1. Which digital media(s) do you have at HOME now?
   You may tick more than one option

Television	☐
Computer (e.g. desktops, laptops)	☐
Mobile devices (e.g. smartphones, tablets)	☐
Video game devices (e.g. console and handheld gaming players, virtual reality headset)	☐
Blu-ray/DVD/CD/Videotape players	☐
Intelligent/ technology toys (e.g. programmable toys)	☐
Others	☐ (please specify): ________________________

***YOUR** digital media habits (all day)*

2. **In the last 7 days,** how much time (in 1 day) do you spend using digital media?
   Indicate N/A where not applicable

Activity	On a typical **weekday**	On a typical **weekend/ public holiday**
Work (e.g. reading documents, checking emails)	___ hr ___ mins	___ hr ___ mins
Entertainment (e.g. watching shows, playing games)	___ hr ___ mins	___ hr ___ mins
Social networking (e.g. browsing social networking sites)	___ hr ___ mins	___ hr ___ mins
Personal development (e.g. seeking for information)	___ hr ___ mins	___ hr ___ mins
Others	___ hr ___ mins (please specify activity): ____________________	___ hr ___ mins (please specify activity): ____________________

SMALLQ® is trademarked in 2018 under Class 41 TM120494
c/o Professor Michael Chia, National Institute of Education, Nanyang Technological University, Singapore.

***YOUR CHILD'S** first exposure to digital media*

3. At what age was your child first exposed to:
   Indicate N/A where not applicable

   (a) fixed screens (television, desktop computer, etc.)
   _______ Year(s) ______ month(s)

   (b) mobile screens (smartphone, tablet, handheld gaming players, etc.)
   _______ Year(s) ______ month(s)

*Digital media habits of **YOUR CHILD outside of kindergarten/childcare centre (e.g. home)***

4. Over the last 7 days, how frequent did your child use screen media?
   Please tick accordingly
   Indicate N/A where not applicable

	Never	1-2 times a week	3-4 times a week	5-6 times a week	7-8 times a week	9 or more times a week	N/A
Television							
Computer (e.g. desktops, laptops)							
Mobile devices (e.g. smartphones, tablets)							
Video game devices (e.g. console and handheld gaming players, virtual reality headset)							
Blu-ray/DVD/CD/Videotape							
Intelligent/ technology toys (e.g. programmable toys)							
Others (please specify): ____________________							

5. Time spent on digital media by **YOUR CHILD outside of kindergarten/childcare centres (e.g. home)**
   In the last 7 days, how much time does your child spend (in 1 day) using digital media?
   Indicate N/A where not applicable

Activity	On a typical **weekday**	On a typical **weekend/ public holiday**
Using media for education/ learning (e.g. reading digital e-book, learning math via educational apps)	___ hr ___ mins	___ hr ___ mins
Using media for entertainment (e.g. watching movies, clips, playing games, listening to music)	___ hr ___ mins	___ hr ___ mins
Creating media (e.g. Creating new apps, programming robots/ interactive toys)	___ hr ___ mins	___ hr ___ mins
Communicating (e.g. chatting with relatives via facetime/ skype)	___ hr ___ mins	___ hr ___ mins
Others	___ hr ___ mins<br>(please specify activity): ____________	___ hr ___ mins<br>(please specify activity): ____________

***YOUR** digital media engagement with your child*

6. When your child uses media, estimate the amount of time you are engaged with him/ her (e.g. interacting with your child while watching videos together)
   Take the total time your child spends using digital media to be 100%

   On a typical **weekday in the last 7 days**: ____ %

   On a typical **weekend/public holiday in the last 7 days**: ____ %

Importance** of digital media use to **YOU AND YOUR CHILD

7. Please indicate how important using digital media is to you and your child:
Please tick accordingly
Indicate N/A where not applicable

**Importance to your child**					
	Not important	Of little importance	Moderately important	Important	Very important
Improve knowledge & skills (e.g. listening, reading, writing, counting)					
Entertainment (e.g. keep my child happy)					
Communication (e.g. chat with relatives via skype)					
**Importance to you**					
	Not important	Of little importance	Moderately important	Important	Very important
Keep child occupied (e.g. when entertaining guests, on long travels)					
Distract or divert child's attention (e.g. coax child to eat during mealtimes, stop child from crying)					
Put child to sleep (e.g. listening to lullaby)					
Others (please specify): ____________________					

***YOUR concerns** with your child's digital media use*

8. How concerned are you with your child's digital media use in relation to:
   Please tick accordingly
   Indicate N/A where not applicable

	Not concerned	Minimally concerned	Somewhat concerned	Concerned	Seriously Concerned
Poor sleep					
Poor eyesight					
Lack of physical exercise and play					
Exposure to inappropriate content (e.g. violence, bad language)					
Addiction (using too much that it affects normal function)					
Lack of parent-child interaction					
Others (please specify): ______________________					

***Guidelines** on digital media use for children*

9. There are guidelines on digital media habits for children. Select the appropriate option:
   Indicate N/A where not applicable

	I am not aware	I am not aware BUT practising	I am aware BUT do not practise	I am aware and practising
Limit digital media use for children younger than 2 years				
Limit screen time to 1 hour per day for children 2-5 years				
Introduce only high-quality educational programs for children 1.5-2 years				
Co-watch or co-play digital media with child (e.g. discuss about what your child and you are watching)				
Others (please elaborate): ______________________				

Segment II: Non-digital behaviour

*Non-digital media habits of **YOUR CHILD outside of kindergarten/childcare centres (e.g. home). We are interested in how your child spends his/her time when NOT using digital media.***

10. In the last 7 days, how much time does your child spend (in a day) in the following activities?
    Indicate N/A where not applicable

Activity	On a typical **weekday**	On a typical **weekend/ public holiday**
Taking afternoon naps	___ hr ___ mins	___ hr ___ mins
Reading printed books	___ hr ___ mins	___ hr ___ mins
Drawing/ painting/ sketching on paper/ others forms of craftwork	___ hr ___ mins	___ hr ___ mins
Helping with simple household chores	___ hr ___ mins	___ hr ___ mins
Indoor play (e.g. dancing, crawling, playing board games, playing with manipulative toys)	___ hr ___ mins	___ hr ___ mins
Outdoor physical play (e.g. playing' hide-and-seek' or 'catching' in a playground	___ hr ___ mins	___ hr ___ mins
Others	___ hr ___ mins<br>(please specify activity):<br>________________	___ hr ___ mins<br>(please specify activity):<br>________________

11. In relation to Question 10, estimate the percentage of time spent on indoor and outdoor physical play that caused your child to breathe faster and harder.
Take the total time your child spends in physical activity to be 100%

On a typical **weekday in the last 7 days**: _____ %

On a typical **weekend/public holiday in the last 7 days**: _____ %

***YOUR** physical play engagement with your child*

12. Estimate the percentage of time you engaged with your child in indoor and outdoor physical play (e.g. playing 'hide-and-seek' or 'catching' together)
Take the total time your child spends in physical play to be 100%

On a typical **weekday in the last 7 days**: _____ %

On a typical **weekend/public holiday in the last 7 days**: _____ %

Segment III: Child and Parent information

Your child information

13. Child's sex: Male ☐ Female ☐

14. Child's age: ________ year(s) ________ month(s)

15. Estimated child's height: _________ cm

    weight: ________ kg

16. Does your child wear spectacles?

    No ☐ Yes ☐

Sleep duration

17. In the last 7 days, what is the average duration of your child's **night-time** sleep?

    Weekday: ________ hour(s) ________ min(s)

    Weekend/ public holiday: ________ hour(s) ________ min(s)

Sleep quality

18. In the last 7 days, how would you rate your child's overall quality of sleep?

    Examples of poor sleep: Wakes up at night, difficulty waking up in the morning, nightmares

    Examples of good sleep: Falls asleep easily at bedtime, uninterrupted sleep

	Unsatisfactory	Below Average	Average	Above Average	Excellent
Weekday					
Weekend/ public holiday					

Parent/ legal guardian information

19. Relationship to child:
    - Mother ☐
    - Father ☐
    - Grandmother ☐
    - Grandfather ☐
    - Legal guardian ☐ (please specify): ________________

20. My age is: ________ year(s) ________ month(s)

21. My estimated height is ________ cm

    weight is ________ kg

22. Estimated monthly household income:
    - $0 - $2500 ☐
    - $2501 - $5000 ☐
    - $5001 - $8000 ☐
    - $8001 - $12000 ☐
    - $12001 - $20000 ☐
    - $20001 and above ☐

23. Highest education attained (parent/ legal guardian completing survey)
    - No formal education ☐
    - Primary school ☐
    - Secondary school ☐
    - Post-secondary institution (JC, ITE, Polytechnic) ☐
    - University - Bachelor degree ☐
    - University - Post-graduate degree (Masters and above) ☐

24. Nationality:

Singapore citizen ☐ Singapore PR ☐ Non-Singapore citizen ☐

25. Ethnicity:

Chinese ☐
Malay ☐
Indian ☐
Eurasian ☐
Others ☐ (Please specify): ____________________

The next segment is Paediatric Quality of Life.

Before proceeding to the final segment, which age group does your child fall under?

☐ 2-4 years ☐ 5-6 years

www.nie.edu.sg
1 Nanyang Walk Singapore 637616 Tel: (65) 6790 3888
NTU Reg No. 200604393R

NATIONAL INSTITUTE OF EDUCATION SINGAPORE

Segment IV: Paediatric Quality of Life

(2-4 years version)

DIRECTIONS

On the following page is a list of things that might be a problem for **your child**.
Please tell us **how much of a problem** each one has been for **your child** during the **past 7 days** by circling:

0 if it is **never** a problem
1 if it is **almost never** a problem
2 if it is **sometimes** a problem
3 if it is **often** a problem
4 if it is **almost always** a problem

There are no right or wrong answers.
If you do not understand a question, please ask for help.

*In the **past 7 days**, how much of a **problem** has your child had with ...*

**PHYSICAL FUNCTIONING *(problems with...)***	**Never**	**Almost Never**	**Some-times**	**Often**	**Almost Always**
1. Walking	0	1	2	3	4
2. Running	0	1	2	3	4
3. Participating in active play or exercise	0	1	2	3	4
4. Lifting something heavy	0	1	2	3	4
5. Bathing	0	1	2	3	4
6. Helping to pick up his or her toys	0	1	2	3	4
7. Having hurts or aches	0	1	2	3	4
8. Low energy level	0	1	2	3	4

**EMOTIONAL FUNCTIONING *(problems with...)***	**Never**	**Almost Never**	**Some-times**	**Often**	**Almost Always**
1. Feeling afraid or scared	0	1	2	3	4
2. Feeling sad or blue	0	1	2	3	4
3. Feeling angry	0	1	2	3	4
4. Trouble sleeping	0	1	2	3	4
5. Worrying	0	1	2	3	4

PedsQL™, Copyright © 1998 JW Varni, Ph.D. All rights reserved.

PedsQL™ contact information and permission to use: Mapi Research Trust, Lyron, France – Internet: https://eprovide.mapi-trust.org and www.pedsql.org/index.html

SMALLQ® is trademarked in 2018 under Class 41 TM120494
c/o Professor Michael Chia, National Institute of Education, Nanyang Technological University, Singapore.

www.nie.edu.sg
1 Nanyang Walk Singapore 637616 Tel: (65) 6790 3888
NTU Reg No. 200604393R

**SOCIAL FUNCTIONING** ***(problems with…)***	**Never**	**Almost Never**	**Some-times**	**Often**	**Almost Always**
1. Playing with other children	0	1	2	3	4
2. Other kids not wanting to play with him/ her	0	1	2	3	4
3. Getting teased by other children	0	1	2	3	4
4. Not able to do things that other children his or her age can do	0	1	2	3	4
5. Keeping up when playing with other children	0	1	2	3	4

**SCHOOL FUNCTIONING** ***(problems with…)***	**Never**	**Almost Never**	**Some-times**	**Often**	**Almost Always**
1. Doing the same school activities with peers	0	1	2	3	4
2. Missing school/ daycare because of not feeling well	0	1	2	3	4
3. Missing school/ daycare to go to the doctor or hospital	0	1	2	3	4

PedsQL™, Copyright © 1998 JW Varni, Ph.D. All rights reserved.

PedsQL™ contact information and permission to use: Mapi Research Trust, Lyron, France – Internet: https://eprovide.mapi-trust.org and www.pedsql.org/index.html

www.nie.edu.sg
1 Nanyang Walk Singapore 637616 Tel: (65) 6790 3888
NTU Reg No. 200604393R

Segment IV: Paediatric Quality of Life

(5-6 years version)

DIRECTIONS

On the following page is a list of things that might be a problem for **your child**.
Please tell us **how much of a problem** each one has been for **your child** during the **past 7 days** by circling:

0 if it is **never** a problem
1 if it is **almost never** a problem
2 if it is **sometimes** a problem
3 if it is **often** a problem
4 if it is **almost always** a problem

There are no right or wrong answers.
If you do not understand a question, please ask for help.

*In the **past 7 days**, how much of a **problem** has your child had with …*

**PHYSICAL FUNCTIONING *(problems with…)***	**Never**	**Almost Never**	**Some-times**	**Often**	**Almost Always**
1. Walking more than one block	0	1	2	3	4
2. Running	0	1	2	3	4
3. Participating in sports activity or exercise	0	1	2	3	4
4. Lifting something heavy	0	1	2	3	4
5. Taking a bath or shower by him or herself	0	1	2	3	4
6. Doing chores, like picking up his or her toys	0	1	2	3	4
7. Having hurts or aches	0	1	2	3	4
8. Low energy level	0	1	2	3	4

**EMOTIONAL FUNCTIONING *(problems with…)***	**Never**	**Almost Never**	**Some-times**	**Often**	**Almost Always**
1. Feeling afraid or scared	0	1	2	3	4
2. Feeling sad or blue	0	1	2	3	4
3. Feeling angry	0	1	2	3	4
4. Trouble sleeping	0	1	2	3	4
5. Worrying about what will happen to him or her	0	1	2	3	4

PedsQL™, Copyright © 1998 JW Varni, Ph.D. All rights reserved.
SMALLQ® is trademarked in 2018 under Class 41 TM120494
c/o Professor Michael Chia, National Institute of Education, Nanyang Technological University, Singapore.

www.nie.edu.sg
1 Nanyang Walk Singapore 637616 Tel: (65) 6790 3888
NTU Reg No. 200604393R

PedsQL™ contact information and permission to use: Mapi Research Trust, Lyron, France – Internet: https://eprovide.mapi-trust.org and www.pedsql.org/index.html

**SOCIAL FUNCTIONING *(problems with…)***	**Never**	**Almost Never**	**Some-times**	**Often**	**Almost Always**
1. Getting along with other children	0	1	2	3	4
2. Other kids not wanting to be his or her friend	0	1	2	3	4
3. Getting teased by other children	0	1	2	3	4
4. Not able to do things that other children his or her age can do	0	1	2	3	4
5. Keeping up when playing with other children	0	1	2	3	4

**SCHOOL FUNCTIONING *(problems with…)***	**Never**	**Almost Never**	**Some-times**	**Often**	**Almost Always**
1. Paying attention in class	0	1	2	3	4
2. Forgetting things	0	1	2	3	4
3. Keeping up with school activities	0	1	2	3	4
4. Missing school because of not feeling well	0	1	2	3	4
5. Missing school to go to the doctor or hospital	0	1	2	3	4

PedsQL™, Copyright © 1998 JW Varni, Ph.D. All rights reserved.

PedsQL™ contact information and permission to use: Mapi Research Trust, Lyron, France – Internet: https://eprovide.mapi-trust.org and www.pedsql.org/index.html

***Impact** of COVID-19 situation:*

Parent's habits

26. **In the last 7 days**, the COVID-19 situation has led to an **increase** in **my** digital media engagement.

(a) Weekday:

Strongly disagree	Disagree	Neutral	Agree	Strongly Agree
1	2	3	4	5

(b) Weekend:

Strongly disagree	Disagree	Neutral	Agree	Strongly Agree
1	2	3	4	5

Child's habits

27. How has your child's average daily time spent on the following activities changed from before COVID-19 compared to the present (think of the last 7 days)?

	Significantly increased	Slightly increased	About the same	Slightly decreased	Significantly decreased
Screen use (computer, smartphone, tablets, TV-viewing)					
Physical activity (indoor & outdoor play, moving from one place to another, playing physical games)					
Outdoor activity (outdoor					

play, exercise or sport)					
Total sleep (inclusive of day-time naps)					
Quality of Life					

- *End of Survey* -

Thank you
